# Progress of research on molecular targeted therapies for colorectal cancer

**DOI:** 10.3389/fphar.2023.1160949

**Published:** 2023-08-08

**Authors:** Shilin Huang, Jiazhou Ye, Xing Gao, Xi Huang, Julu Huang, Lu Lu, Cheng Lu, Yongqiang Li, Min Luo, Mingzhi Xie, Yan Lin, Rong Liang

**Affiliations:** ^1^ Department of Digestive Oncology, Guangxi Medical University Cancer Hospital, Nanning, China; ^2^ Department of Hepatobiliary Surgery, Guangxi Medical University Cancer Hospital, Nanning, China

**Keywords:** colorectal cancer, EGFR, HER2, BRAF, anti-angiogenic

## Abstract

Colorectal cancer (CRC) is one of the most common malignancies, accounting for approximately 10% of global cancer incidence and mortality. Approximately 20% of patients with CRC present metastatic disease (mCRC) at the time of diagnosis. Moreover, up to 50% of patients with localized disease eventually metastasize. mCRC encompasses a complex cascade of reactions involving multiple factors and processes, leading to a diverse array of molecular mechanisms. Improved comprehension of the pathways underlying cancer cell development and proliferation, coupled with the accessibility of relevant targeted agents, has propelled advancements in CRC treatment, ultimately leading to enhanced survival rates. Mutations in various pathways and location of the primary tumor in CRC influences the efficacy of targeted agents. This review summarizes available targeted agents for different CRC pathways, with a focus on recent advances in anti-angiogenic and anti-epidermal growth factor receptor agents, BRAF mutations, and human epidermal growth factor receptor 2-associated targeted agents.

## 1 Introduction

Colorectal cancer (CRC) is the third most common cancer and the third leading cause of cancer-related death worldwide. The onset of CRC is subtle and challenging to detect in early stages, underscoring the importance of timely screening. Moreover, approximately 22% of patients are diagnosed with metastatic disease at the outset. The 5-year metastatic CRC (mCRC) survival rate is approximately 15% (Howlader N et al., 2020). Therefore, precise treatment of mCRC using appropriate drugs based on relevant molecular biosignatures is essential to prolong the survival time of patients.

**FIGURE 1:**
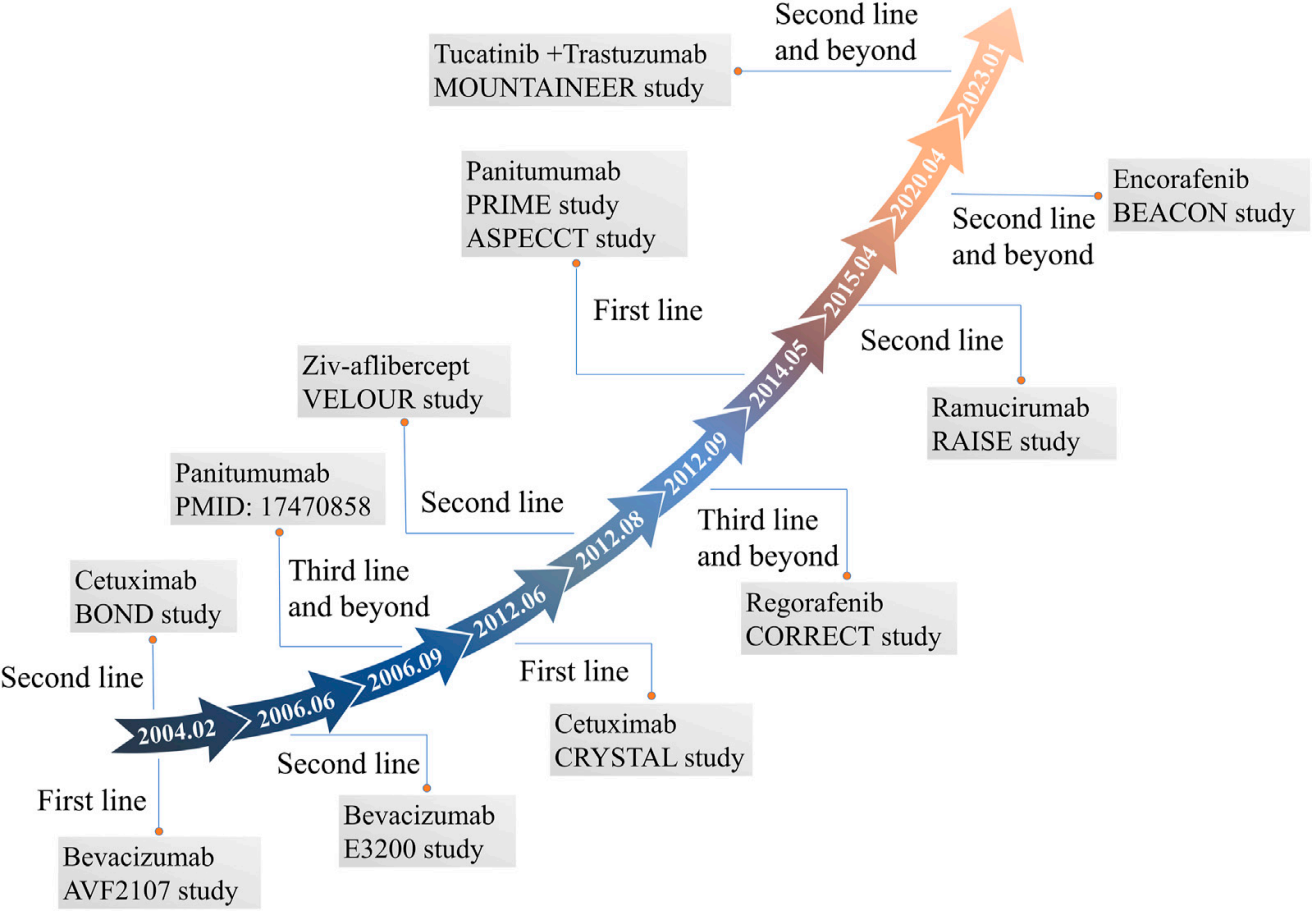
FDA-approved molecular targeted agents for mCRC and their associated clinical studies.

The main treatment strategies for mCRC are chemotherapy using cytotoxic agents, molecular-targeted therapy, and immunotherapy. Combination chemotherapy regimens frequently involve the use of fluorouracil (5-FU)-based drugs together with oxaliplatin or irinotecan. These combinations have demonstrated significant improvements in patient survival, with survival durations exceeding 20 months (Tournigand et al., 2004; Van Cutsem et al., 2014; García-Alfonso et al., 2021; Glimelius et al., 2021). Rapid and significant advancement in the development of targeted agents for CRC has been observed since the approval of cetuximab for mCRC treatment in 2004 (Figure 1) Currently, more than 10 agents have been approved for mCRC treatment. Combined treatments utilizing targeted drugs and chemotherapy have been reported to increase the 5-year survival rate from 9% to 15% and survival to more than 30 months in mCRC patients (Van Cutsem et al., 2016; Siegel et al., 2022). As distant metastasis is the main cause of death in CRC, relevant review that comprehensively explores and summarizes the efficacy of molecularly targeted drugs in CRC and associated latest research progress does not exist, and this review highlights these. This review focuses on progress of research on targeted agents for treating mCRC patients, providing a reference for clinicians for precise treatment of CRC.

## 2 Classification and mechanism of common targets in CRC

### 2.1 Vascular endothelial growth factor (VEGF) target

**FIGURE 2:**
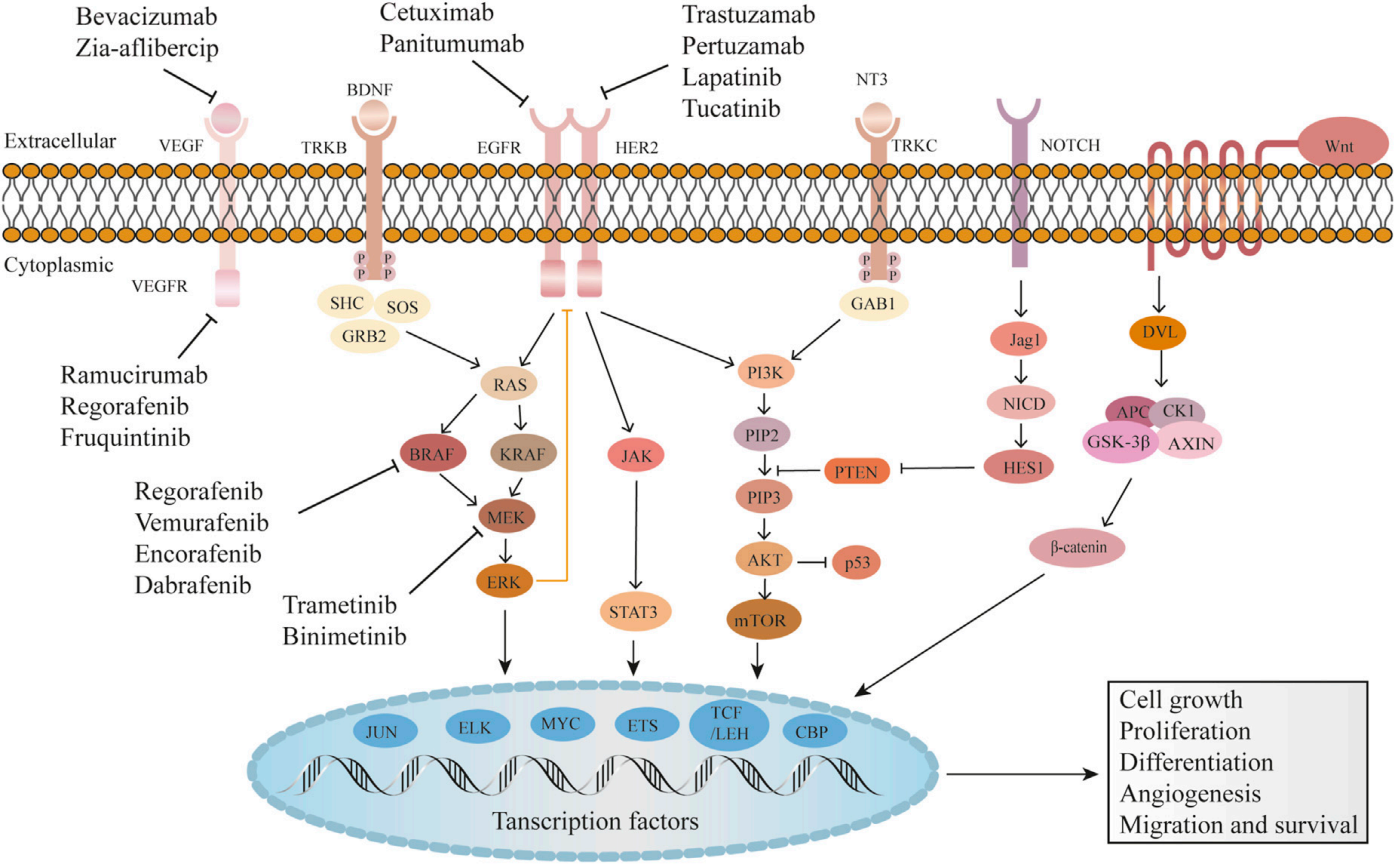
Common molecular targets and their mechanisms of action in CRC.

VEGF family includes VEGFA–D, VEGF receptor (VEGFR), and placental growth factor. VEGFA, commonly known as VEGF or vascular permeability factor, is the main angiogenic feature of the VEGF family. The role of VEGFB in tumor angiogenesis has not yet been elucidated; however, VEGFC and VEGFD mainly regulate lymphatic endothelial cell growth (Dvorak, 2002; Karkkainen et al., 2002; Ferrara et al., 2003; Bry et al., 2014). VEGFR includes VEGFR1–3, with VEGFA capable of binding to VEGFR1 and 2, and VEGFC and VEGFD binding to VEGFR2 and 3, respectively. VEGFR activation can promote cell proliferation, migration, and growth via the mitogen-activated protein kinase (MAPK) and phosphoinositide 3-kinases (PI3K) pathways, leading to angiogenesis and tumor angiogenesis (Ivy et al., 2009). VEGF inhibitors used in CRC treatment include monoclonal antibodies (mAbs) like bevacizumab, ramucirumab, Ziv-aflibercept, as well as tyrosine kinase inhibitors (TKIs), such as sunitinib, sorafenib, fruquintinib, and regorafenib. These drugs target the VEGF pathway to inhibit angiogenesis and tumor growth (Figure 2).

### 2.2 EGFR and HER2 target

Epidermal growth factor receptor (EGFR), also known as ERBB1 or human epidermal growth factor receptor (HER) 1, is a member of the HER family, which also includes HER2–4. EGFR promotes tumor cell proliferation, differentiation, growth, and distant metastasis by activating downstream signaling pathways, such as PI3K and MAPK (Roskoski, 2014; Kumagai et al., 2021). EGFR activation induces the secretion of angiogenic factors, such as VEGF, which stimulate the formation of new blood vessels. Increased angiogenesis ensures sufficient blood supply to the tumor, facilitating its expansion and providing nutrients for sustained growth. Additionally, EGFR signaling provides anti-apoptotic signals through the activation of AKT, which inhibits apoptosis by inactivating pro-apoptotic proteins (De Luca et al., 2008). This allows cancer cells to evade programmed cell death and survive in unfavorable conditions. Furthermore, HER2 overexpression, as a member of the EGFR family, inhibits the tumor suppressor gene, p53 (Ménard et al., 2003). Common EGFR inhibitors used in cancer treatment include anti-EGFR mAbs, such as cetuximab and panitumumab, EGFR TKIs like gefitinib, erlotinib, and afatinib, as well as HER2 mAbs trastuzumab and pertuzumab, and HER2 TKI lapatinib. The inhibitors target EGFR and HER2, blocking their signaling pathways and inhibiting tumor growth and progression.

### 2.3 RAS/RAF/MEK/ERK target

The RAS/RAF/MEK/ERK signaling cascade (MAPK) pathway is one of the downstream EGFR pathways, which mainly regulates cell proliferation and differentiation. RAS family members include KRAS, NRAS, and HRAS (Karnoub and Weinberg, 2008). KRAS mutation (MT) accounts for approximately 32%–40% of CRC cases, and BRAF V600E MT accounts for 5%–15% (De Roock et al., 2011; Pakneshan et al., 2013). MEK, or MAPK/ERK kinase, plays a pivotal role as a mediator in the downstream signaling of the MAPK pathway. ERK, also known as extracellular signal-regulated kinase, is a critical component of this pathway (Downward, 2003). Studies have confirmed that MEK/ERK inhibitors significantly enhance the treatment efficacy in patients with KRAS/BRAF-mutant tumors (Hatzivassiliou et al., 2013; Morris et al., 2013). Available inhibitors targeting the RAS/RAF/MEK/ERK pathway include dabrafenib and vemurafenib as BRAF inhibitors, and trametinib and encorafenib as MEK inhibitors. Additionally, there are ERK inhibitors such as ulixertinib and temuterkib.

### 2.4 PI3K/AKT/mTOR target

PI3K is an enzyme that plays a crucial role in cellular signaling pathways involved in cell growth, survival, and metabolism. It functions by phosphorylating the lipid phosphatidylinositol 4,5-bisphosphate (PIP2) to generate phosphatidylinositol 3,4,5-trisphosphate (PIP3), which in turn activates downstream signaling pathways. PIP3 acts as a docking site for AKT, facilitating its activation through phosphorylation. Notably, one of the significant downstream AKT targets the mammalian target of rapamycin (mTOR), which plays a critical role in protein synthesis, cellular growth, and metabolic regulation. Aberrant activation of PI3K can occur through various mechanisms, including genetic mutations, PI3K genes amplification, or upstream receptors activation such as EGFR. Dysregulation of the PI3K/AKT/mTOR pathway has been implicated in various diseases, including cancer, highlighting its significance as a therapeutic target for intervention and treatment strategies (Fruman and Rommel, 2014; Polivka and Janku, 2014; Janku et al., 2018). Several inhibitors have been developed to target the PI3K/AKT/mTOR pathway. These include PI3K inhibitors such as buparlisib and sonolisib, which specifically target the PI3K enzyme and AKT inhibitors like MK-2206 and ipatasertib that block the activity of the AKT protein, a downstream effector of PI3K. Sirolimus and everolimus are targeted towards mTOR, a key component of the pathway. Additionally, dual PI3K/mTOR inhibitors such as dactolisib and apitolisib simultaneously target PI3K and mTOR.

### 2.5 NOTCH target

NOTCH signaling involves the activation of NOTCH receptors by ligands such as JAG1, resulting in the release of the Notch intracellular domain (NICD). The NICD then translocates to the nucleus and forms a complex with transcriptional regulators to activate target genes, including the hairy and enhancer of split (HES) family genes. This signaling pathway plays a crucial role in cell fate determination, differentiation, and various cellular processes (Siebel and Lendahl, 2017; Li et al., 2023). NOTCH receptors mAbs such as tarextumab and demcizumab, have been developed and tested in preclinical and clinical studies.

### 2.6 Wnt target

The Wnt signaling pathway plays a critical role in various biological processes, including embryonic development, tissue homeostasis, and cell proliferation. Dysregulation of this pathway has been implicated in several diseases, particularly cancer. Recruitment and activation of Disheveled (Dvl) are initiated by the activation of the Wnt signaling pathway. Subsequently, a complex involving Adenomatous Polyposis Coli (APC), glycogen synthase kinase 3β (GSK-3β), Casein kinase 1 (CK1), and Axin forms, leading to phosphorylation and inhibition of GSK3β (Duchartre et al., 2016; Zhang and Wang, 2020). This, in turn, results in increased levels of β-catenin, a pivotal component of the Wnt signaling pathway, which drives cancer cell proliferation. WNT974 and CGX1321, inhibitors targeting the Wnt ligand/receptor interface, have shown promising efficacy in preclinical studies (Rodon et al., 2021a; Rodon et al., 2021b).

### 2.7 NTRK gene fusion target

The tropomyosin receptor kinase (TRK) family comprises TRK A–C, which is encoded by neurotrophic TRK (NTRK)1–3. NTRK gene fusion occurs when the 3′region of the NTRK gene and 5′end of the fusion chaperone gene are connected by intra-chromosome or inter-chromosome rearrangement. The protein encoded by the fusion gene can bind to TRK and activate the downstream PI3K and MAPK pathways, resulting in tumor growth, proliferation, and differentiation. NTRK gene fusion was first identified in patients with CRC and then in those with other tumors (Martin-Zanca et al., 1986; Vaishnavi et al., 2015; Amatu et al., 2019; Solomon et al., 2019). Entrectinib, which targets the NTRK fusion gene, has been shown to be highly therapeutic for patients with NTRK. (2019).

## 3 Common targeted agents for CRC therapy

### 3.1 Bevacizumab

Bevacizumab specifically binds to VEGF, blocking its interaction with the receptor, degrading existing tumor blood vessels, normalizing surviving ones, and inhibiting tumor neovascularization to exert antitumor effects.

#### 3.1.1 Adjuvant therapy

The QUASAR 2 study showed that CRC patients treated with capecitabine (Cap) + bevacizumab or only Cap after radical (R0) resection had 3-year disease-free survival (DFS) rates of 75.4% and 78.4%, respectively (Kerr et al., 2016). Similarly, the NSABP protocol C-08 and other studies (Allegra et al., 2011; de Gramont et al., 2012; André et al., 2020) confirmed that neoadjuvant chemotherapy with bevacizumab in combination with oxaliplatin did not increase DFS in patients compared to that in patients treated with chemotherapy alone. Overall, none of these trials showed a significant survival benefit for CRC patients treated with adjuvant bevacizumab; therefore, none of the current guidelines recommend the use of bevacizumab as adjuvant therapy. Adjuvant and neoadjuvant treatment trials in mCRC are summarized in Table 1.

**TABLE 1:** Adjuvant and neoadjuvant treatment trials in mCRC.

Trial name	Clinical number	Patients (n)	Population	Treat line	Treatment	Result	DFS (%)		HR		OS (%)
							3 years	5 years	*p*-value		5 years
NSABP protocol C-08	NCT00096278	2710	NA	Adjuvant therapy	FOLFOX6 +Bev	negative	77.4				
					FOLFOX6		75.5				
AVANT	NCT00112918	3451	NA	Adjuvant therapy	FOLFOX4 +Bev	negative	73.0				
					CAPOX + Bev		75.0				
					FOLFOX4		76.0				
S-AVANT	NCT00112918	2867	NA	Adjuvant therapy	FOLFOX4 +Bev	negative			68.5		
					CAPOX + Bev				71.0		
					FOLFOX4				3.2		
QUASAR 2	ISRCTN45133151	1952	NA	Adjuvant therapy	CAP + Bev	negative	75.4				80.0
					CAP		78.4				
E3204	NCT00321685	57	NA	Neoadjuvant therapy + Adjuvant therapy	CAPOX + Bev + Radiotherapy (Adjuvant therapy)	positive			81		83.7
					FOLFOX + Bev (Neoadjuvant therapy)						88.3
ECOG-ACRIN-E5204	NCT00303628	355	NA	Adjuvant therapy	mFOLFOX6 +Bev	negative			76.5		
					mFOLFOX6				71.2		
CTRUST	NCT03085992	49	NA	Neoadjuvant therapy	FOLFOXIRI + Bev	positive	80.45 (2 years)				72.2
					Radiotherapy + Bev						
					Surgery						
CRAB	NCT00842686	61	NA	Neoadjuvant therapy	CAP + Bev + Radiotherapy	positive			70.0		
[NCCTG] N0147	NCT00079274	2686	KRAS WT	Adjuvant therapy	FOLFOX6+Cet	negative	71.5			1.21	
							74.6			*p* = 0.08	
			KRAS MT		FOLFOX6		67.1			1.12	
							65.0			*p* = 0.38	
EXPERT-C		165	KRAS/BRAF	Neoadjuvant therapy	Radiotherapy after CAPOX + Cet Radiotherapy after CAPOX	NA					27.0
											*p* < 0.034
PETACC-8	EudraCT, number 2005-003463-23	2559	KRAS WT	Adjuvant therapy	FOLFOX4+Cet	negative	75.1			1.05	
							79.1			*p* = 0.66	
			KRAS/BRAF WT				75.9			0.99	
					FOLFOX4		79.1			*p* = 0.92	
			KRAS MT				70.7			1.06	
							71.0			*p* = 0.65	
SWOG 0713	NCT00686166	83	KRAS WT	Neoadjuvant therapy	Radiotherapy and CAPOX + Cet after CAPOX-Cet	NA	72.0				

Abbreviations: DFS, disease-free survival; OS, overall survival; Cet, cetuximab; Bev, bevacizumab; CAP, capecitabine; NA, not applicable; WT, mutation.

#### 3.1.2 Neoadjuvant therapy

A previous study showed that six cycles of bevacizumab in combination with chemotherapy, followed by chemoradiotherapy (CRT) achieved an objective response rate (ORR) of 88.9% in locally advanced colorectal patients with T4 or high-risk T3, with R0 resection rate of 97.8% (Masi et al., 2019). Similarly, the CRAB study reported a 95% R0 resection rate in patients with stage II/III rectal cancer treated with neoadjuvant bevacizumab + CRT (Velenik et al., 2020). Although these studies reported positive outcomes, available evidence is insufficient to support the adoption of bevacizumab as a standard neoadjuvant (Table 1).

#### 3.1.3 First-line treatment

The AVF2107 study showed the administration of FOLFIRI + bevacizumab as a first-line treatment significantly improved (*p* < 0.001) the median overall survival (mOS; 20.3 vs. 15.6 months) and median progression-free survival (mPFS; 10.6 vs. 6.2 months) of mCRC patients compared to those of the mCRC patients treated with chemotherapy alone (Hurwitz et al., 2004). This finding facilitated the approval of bevacizumab by the United States Food and Drug Administration (FDA) as a first-line mCRC treatment in 2004. Additionally, the administration of bevacizumab + chemotherapy significantly increased (*p* = 0.0023) the mPFS of patients compared to that of the patients treated with chemotherapy alone (9.4 vs. 8.0 months) but did not affect mOS (Saltz et al., 2008). Moreover, both the MAVERICC (Parikh et al., 2019) and ARIES studies (Bendell et al., 2012) confirmed that FOLFIRI or FOLFOX + bevacizumab had similar PFS and OS.

However, the TRIBE2 study (Cremolini et al., 2020) showed that FOLFOXIRI + bevacizumab achieved higher mOS (27.4 vs. 22.5 months) and mPFS (19.2 vs. 16.4 months) than mFOLFOX6 + bevacizumab. The treatment benefit was independent of RAS and BRAF mutation status but was better in patients with right-sided tumors, and the same benefit was achieved in patients who progressed after treatment with FOLFOXIRI + bevacizumab (Loupakis et al., 2015; Cremolini et al., 2018b). Moreover, both the AVEX (Cunningham et al., 2013) and PRODIGE20 (Aparicio et al., 2018a) studies confirmed that bevacizumab provided increased treatment benefits in elderly patients and did not induce adverse events. Key trials of anti-VEGF agents in the treatment of mCRC are illustrated in Table 2.

**TABLE 2:** Key trials of anti-VEGF agents in the treatment of mCRC.

Trial name	Clinical number	Patients (n)	Treat line	Treatment	Result	mOS (months)	HR	mPFS (months)	HR	ORR (%)
							*p*-value		*p*-value2	
AVF2107	NCT00109070	813	First line	FOLFIRI + Bev	Positive	20.3	0.66	10.6	0.54	
				FOLFIRI + placebo		15.6	*p* < 0.001	6.2	*p* < 0.001	
ITACa	NCT01878422	376	First line	FOLFIRI/FOLFOX + Bev	Positive	9.6	0.86	20.8	1.13	
				FOLFIRI/FOLFOX		8.4	*p* = 0.182	21.3	*p* = 0.317	
NO1966	NCT00069095	1401	First line	CAPOX/FOLFOX4 + Bev	Positive	21.3	0.89	9.4	0.83	
				CAPOX/FOLFOX4 + placebo		19.9	*p* = 0.077	8.0	*p* = 0.0023	
MAVERICC	NCT01374425	376	First line	mFOLFOX6 + Bev	Negative	10.1	0.79	23.9	0.76	
				FOLFIRI + Bev		12.6	*p* = 0.06	27.5	*p* = 0.09	
TRIBE	NCT00719797	508	First line	FOLFOXIRI + Bev	Positive	31.0	0.79	12.1	0.75	65.0
				FOLFIRI + Bev		25.8	*p* = 0.054	9.7	*p* = 0.003	53.0
TRIBE2	NCT02339116	679	First line	mFOLFOX6 + Bev	Positive	27.4	0.82	19.2	0.74	62.0
				FOLFOXIRI + Bev		22.5	*p* = 0.032	16.4	*p* = 0.0005	50.0
TRIBE2	NCT02339116	679	Second line	FOLFOXIRI	Positive			12.0	0.74	
				FOLFIRI				9.8	*p* = 0.0002	
AVEX	NCT00484939	280	First line	CAP + Bev	Positive	20.7	0.79	9.1	0.53	
				CAP		16.8	*p* = 0.18	5.1	*p* < 0.0001	
PRODIGE 20	NCT01900717	102	First line	chemotherapy + Bev	Positive	21.7	0.73	9.7	0.79	37.2
				chemotherapy		19.8		7.8		32.6
PRODIGE 9	NCT00952029	491	Maintenance after First line	FOLFOX + Bev	Negative	21.7	1.07	9.2	0.91	
				Observation		22.0	*p* = 0.500	8.9	*p* = 0.316	
AIO 0207	NCT00973609	837	Maintenance after First line	Fol + Bev	Positive	20.2	*p* = 0.77	6.3	*p* < 0.0001	
				Bev		21.9		4.6		
				Observation		23.2		3.5		
CAIRO3	NCT00442637	558	Maintenance after First line	CAP + Bev	Positive	25.9	0.89	8.5	0.43	
				Observation		22.4	*p* = 0.22	4.1	*p* ≤ 0.0001	
CAIRO3	NCT00442637	558	Second line	CAP + Bev (progress after maintenence)	Positive			11.7	0.67	
								8.5	*p* ≤ 0.0001	
E3200	NCT00025337	829	Second line	FOLFOX4 + Bev	Positive	12.9	0.75	7.3	0.61	
				FOLFOX4		10.8	*p* = 0.0011	4.7	*p* < 0 .0001	
				Bev		10.2		2.7		
ML18147	NCT00700102	820	Second line	chemotherapy + Bev	Positive	11.2	0.81	5.7	0.68	
				chemotherapy		9.6	*p* = 0.0062	4.1	*p* < 0.0001	
BEBYP	NCT00720512	185	Second line	chemotherapy + Bev	Positive	14.1	0.77	6.8	0.70	
				chemotherapy		15.5	*p* = 0.043	5.0	*p* = 0.010	
ARIES	NCT00388206	1550	First line	FOLFOX + BEV	NA	23.7	0.95	10.3	1.03	
				FOLFIRI + BEV		25.5	*p* = 0.625	10.2	*p* = 0.688	
ARIES	NCT00388206	482	Second line	BEV + chemotherapy (first-line Bev-exposed VS. first-line Bev-naive)	Positive	19.8		7.6		
						17.2		8.1		
BRiTE		1445	Second line	BEV + chemotherapy (first-line Bev-exposed VS. first-line Bev-naive)	Positive	19.9	0.48			
						31.8	*p* < 0 .001			
Pfeiffer et al. (2020)	EudraCT, 2016–005241–23	93	Third line and beyond	TAS-102 + Bev	Positive	9.4	0.55	4.6	0.45	
				TAS102		6.7	*p* = 0.028	2.6	*p* = 0.0015	
TAS-CC3	UMIN000022438	32	Third line and beyond	TAS-102 + Bev	Positive	4.5		9.2		
AFFIRM	NCT00851084	236	First line	mFOLFOX6 + Ziv-aflibercept	Negative	19.5	0.98	8.48	1	
				mFOLFOX6		22.3		8.77		
VELOUR	NCT00561470	1226	Second line	FOLFIRI + Ziv-aflibercept	Positive	13.5	0.817	6.90	0.758	
				FOLFIRI		12.06	*p* = 0.0032	4.67	*p* < 0.0001	
RAISE	NCT01183780	1072	Second line	Ramucirumab + FOLFIRI	Positive	13.3	0.844	5.7	0.793	
				Ramucirumab		11.7	*p* = 0.0219	4.5	*p* < 0.0005	
De Gramont et al. (2012)	NCT01111604	153	Second line	mFOLFOX-6 + Ramucirumab	Negative	41.7 w	1.18	21.4 w	1.116	
				mFOLFOX-6		53.6 w		18.4 w	*p* = 0.623	

mOS, median overall survival; mPFS, median progression-free survival; ORR, objective response rate; Bev, bevacizumab; CAP, capecitabine; TAS-102, trifluridine and tipiracil; NA, not applicable; WT, mutation.

#### 3.1.4 Maintenance treatment

In the CAIRO3 study (Simkens et al., 2015), bevacizumab + Cap was used as maintenance treatment for mCRC patients after first-line treatment with chemotherapy + bevacizumab that led to improved mPFS (8.5 vs. 4.1 months, *p* < 0.0001); however, the maintenance treatment did not affect OS. Similarly, both the PRODIGE9 (Aparicio et al., 2018b) and AIO0207 (Hegewisch-Becker et al., 2015) studies confirmed bevacizumab-induced improvement in PFS after induction chemotherapy. Moreover, the 2016 ESMO guidelines state that Cap + bevacizumab can be used for maintenance therapy after first-line treatment, but bevacizumab alone is not recommended for maintenance therapy. Additionally, the National Comprehensive Cancer Network (NCCN) guidelines do not recommend bevacizumab as maintenance therapy.

#### 3.1.5 Second-line treatment

The E3200 study (Giantonio et al., 2007) showed that the administration of bevacizumab as a second-line treatment in mCRC patients achieved mOS of 12.9, 10.2, and 10.8 months and mPFS of 7.3, 2.7, and 4.7 months in patients subjected to FOLFOX + bevacizumab, bevacizumab only, or chemotherapy only first-line therapies, respectively. Thus, bevacizumab was approved in 2006 as a second-line treatment for patients with mCRC. Additionally, the ML18147 study showed that the administration of chemotherapy + bevacizumab or chemotherapy only as a second-line treatment achieved mOS of 11.2 vs. 9.6 months (*p* = 0.0062) and mPFS of 5.7 vs. 4.1 months (*p* < 0.0001), respectively, in mCRC patients subjected to bevacizumab as a first-line therapy (Bennouna et al., 2013). Moreover, the ML18147 and several other studies confirmed that retreatment with bevacizumab-containing regimens exerted significant therapeutic effects in mCRC patients who received bevacizumab-containing regimens as first-line therapy (Grothey et al., 2008; Hurwitz et al., 2014). Based on these findings, the FDA approved bevacizumab as a cross-line therapy in 2013.

#### 3.1.6 Third-line treatment and beyond

A previous study showed that trifluridine and tipiracil (TAS-102) + bevacizumab was more effective than TAS-102 alone as a third-line treatment in patients with anti-EGFR positive and chemotherapy-resistant mCRC, with considerable increase in mOS (9.4 vs. 6.7 months, *p* = 0.028) and mPFS (4.6 vs. 2.6 months, *p* = 0.0010) (Pfeiffer et al., 2020). Similarly, the TAS-CC3 study confirmed the positive effects of TAS-102 + bevacizumab as a third-line treatment in Asian patients with mCRC (Yoshida et al., 2021). Accordingly, the NCCN guidelines recommend the use bevacizumab in combination with TAS-102 as a third-line treatment in patients with mCRC (Table 2).

### 3.2 Ziv-aflibercept

Ziv-aflibercept is an anti-VEGF agent that inhibits neovascularization by tightly binding to VEGF and reducing vascular permeability. The AFFIRM study showed (Folprecht et al., 2016) that abatacept did not achieve promising outcomes as a first-line treatment for mCRC. However, considerable therapeutic benefit was obtained with Ziv-aflibercept as a second-line treatment (Van Cutsem et al., 2012). Notably, FOLFIRI + Ziv-aflibercept was more effective than FOLFIRI only as a second-line treatment in mCRC patients, with improved mOS (13.5 vs. 12.06 months, *p* = 0.0032) and mPFS (6.90 vs. 4.67 months, *p* < 0.0001). Accordingly, Ziv-aflibercept was approved by the FDA as a second-line treatment for mCRC patients who progressed or were resistant to first-line oxaliplatin therapy in 2012 (Table 2).

### 3.3 Cetuximab

Cetuximab binds specifically to EGFR and competitively blocks VEGF and other receptors, inhibiting intracellular signaling pathways, thereby suppressing the proliferation of cancer cells and inducing apoptosis.

#### 3.3.1 Adjuvant therapy

In the N0147 [NCCTG] study (Alberts et al., 2012), mFOLFOX + cetuximab did not show significant health-promoting effects as an adjuvant compared to those of chemotherapy alone, with no statistical difference in 3-year DFS in patients with KRAS wild-type (WT). Moreover, the PETACC-8 (Taieb et al., 2014) study confirmed that cetuximab adjuvant therapy had no survival benefit in patients with KRAS WT. Current guidelines do not recommend the use of cetuximab as an adjuvant therapy (Table 1).

#### 3.3.2 Neoadjuvant treatment

The EXPERT-C study (Dewdney et al., 2012) showed that CAPOX + cetuximab was more effective than CAPOX only (four cycles each) in neoadjuvant therapy, with significantly higher response rate (RR) and OS in the cetuximab group. However, some studies, including the COIN study (Maughan et al., 2011; Tveit et al., 2012), showed that cetuximab treatment did not improve patient survival; in contrast, the TAILOR study (Qin et al., 2018) confirmed cetuximab OS benefit. The 2012 NCCN guidelines do not recommend cetuximab as a neoadjuvant therapy because of insufficient evidence (Table 1).

#### 3.3.3 First-line treatment

The CRYSTAL study (Van Cutsem et al., 2009) showed that treatment with FOLFIRI + cetuximab significantly increased mOS (24.9 vs. 21.0 months) and mPFS (9.9 vs. 8.7 months) in patients with KRAS WT mCRC compared to those in patients with KRAS WT treated with FOLFIRI treatment alone. Accordingly, cetuximab was approved by the FDA in 2012 as a first-line treatment in patients with KRAS WT mCRC. In contrast, the COIN (Maughan et al., 2011) and NORDIC-VII (Tveit et al., 2012) studies showed insignificant differences in OS and PFS between oxaliplatin + cetuximab and oxaliplatin treated patients with KRAS WT mCRC. However, the TAILOR study (Qin et al., 2018) showed that cetuximab + FOLFOX increased mPFS (9.2 vs. 7.4 months, *p* = 0.004) and mOS (20.7 vs. 17.8 months, *p* = 0.02) in patients with RAS WT mCRC compared to those in FOLFOX alone treated patients with RAS WT mCRC. Moreover, previous studies have shown that cetuximab was as effective as bevacizumab in patients with RAS WT (Heinemann et al., 2014; Venook et al., 2017). Additionally, the NCT00208546 study showed insignificant difference in efficacy between cetuximab + bevacizumab and cetuximab only (Tol et al., 2009); therefore, cetuximab + bevacizumab is not recommended for patients with mCRC.

The CALGB/SWOG80405 (Alliance) study (Venook et al., 2017) found that cetuximab achieved significantly higher OS and PFS in patients with left-sided primary tumors than in those with right-sided primary tumors. Several studies confirmed that anti-EGFR antibody in combination with chemotherapy exerted the best effects in patients with left-sided primary tumors and RAS WT mCRC (Arnold et al., 2017; Tejpar et al., 2017). Pivotal trials of anti-EGFR monoclonal antibody treatment in mCRC are summarized in Table 3.

**TABLE 3:** Pivotal trials of anti-EGFR mAbs treatment in mCRC.

Trial name	Clinical number	Patiens (n)	Population	Treat line	Treatment	Result	mOS (months)	HR	mPFS (months)	HR	ORR (%)
								*p*-value		*p*-value	
CRYSTAL	NCT00154102	599	EGFR (+)	First-line	FOLFIRI + Cet	Positive	ITT: 19.9	0.93	ITT: 8.9	0.85	
							18.6	*p* = 0.31	8.0	*p* = 0.048	
					FOLFIRI		KRAS WT: 24.9	0.84	KRAS WT: 9.9	0.68	
							21.0		8.7	*p* = 0.02	
COIN	NCT00182715	1630	NA	First-line	CAPOX/FOLFOX + Cet	Negative	17.0	1.04	8.6	0.96	
					CAPOX/FOLFOX		17.9	*p* = 0.67	8.6	*p* = 0.60	
TAILOR	NCT01228734	393	RAS WT	First-line	FOLFOX4 + Cet	Positive	20.7	0.69	9.2	0.69	61.1
					FOLFOX-4		17.8	*p* = 0.004	7.4	*p* = 0.004	39.5
NORDIC-VII	NCT00145314	566	NA	First-line	FOLFOX	Negative	20.4	0.89	7.9		41.0
					FOLFOX + Cet		19.7	*p* = 0.31	8.3		49.0
					FOLFOX (intermittent)+ Cet		20.3	(First two groups)	7.3		47.0
GALGB/SWOG	NCT00265850	1137	KRAS WT	First-line	FOLFOX/FOLFIRI + Cet	Negative	30.0	0.88	10.5	0.95	
					FOLFOX/FOLFIRI + Bev		29.0	*p* = 0.08	10.6	*p* = 0.45	
FIRE3	NCT00433927	400	NA	First-line	FOLFIRI + Cet	Positive	ITT: 28.7	0.77	ITT: 10.0	1.06	
							25.0	*p* = 0.017	10.3	*p* = 0.55	
							RAS WT: 31.1	0.70	RAS WT: 10.4		
					FOLFIRI + Bev		25.6	*p* = 0.011	10.2	*p* = 0.54	
Tol et al. (2009)	NCT00208546	755	NA	First-line	CAPOX + Cet	Negative	20.3	*p* = 0.16	10.7	1.22	
					CAPOX + Bev + Cet		19.4		9.4		
MACRO2 TTD	NCT01161316	193	KRAS WT	Maintenance after First-line	Cet alone	Negative	23.0	1.2	9.0	1.2	
					mFOLFOX6 + Cet		27.0	*p* = 0.2649	10.0	*p* = 0.3907	
Wang et al. (2022)	NCT02717923	47	RAS WT	Maintenance after First-line	Cap + Cet	NA	27.4		12.7		
MACBETH	NCT02295930	143	RAS/BRAF WT	Maintenance after First-line	Cet	NA			13.3	0.73	
					Bev				10.8		
BOND		329	EGFR (+)	Second-line	IRI + Cet	Positive	4.1	*p* < 0.001	8.6	*p* = 0.48	
					Cet		1.5		6.9		
CAPRI-GOIM	EudraCT number 2009-014041-81	153	KRAS WT	Second-line	FOLFOX + Cet	Positive	23.7	0.57	6.9	0.56	
					FOLFOX		19.8	*p* = 0.056	5.3	*p* = 0.025	
EPIC		1298	EGFR (+)	Second-line	Cet + IRI	Positive	10.7	0.975	4.0	0.692	
					IRI		10.0	*p* = 0 .71	2.6	*p* < 0.0001	
The UNICANCER PRODIGE18	NCT01442649	132	KRAS WT	Second-line	chemotherapy + Bev	NA	15.8	0.69	7.1	0.71	24.6
					chemotherapy + Cet		10.4	*p* = 0.08	5.6	*p* = 0 .06	31.8
Jonker DJ	NCT00079066	572	EGFR (+)	Third-line and beyond	BSC + Cet	Positive	6.1	0.77		0.68	
					BSC		4.6	*p* = 0.005		*p* < 0.001	
Vincenzi B		55	EGFR (+)	Third-line and beyond	IRI + Cet	NA	9.8		4.7		
Santini D		39	KRAS WT	Third-line and beyond (Rechallenge)	IRI + Cet	NA			6.6		
JACCRO CC-08	UMIN000010638	34	KRAS WT	Third-line and beyond (Rechallenge)	IRI + Cet	NA	8.2		2.4		
PRIME	NCT00364013	1183	NA	First-line	FOLFOX4 + Pmab	Positive	RAS WT: 23.9	0.83	RAS WT: 9.6	0.8	
							19.7	*p* = 0.072	8.0	*p* = 0.02	
					FOLFOX4		RAS MT: 15.5	1.2	RAS MT: 7.3	1.29	
							19.3	*p* = 0.068	8.8	*p* = 0 .02	
ASPECCT	NCT01001377	1010	KRAS WT	Third-line	Pmab + BSC	NA	10.4	0.97	4.4	1	
					Cet + BSC		10.0		4.1		
PEAK	NCT00819780	285	KRAS WT	First-line	mFOLFOX6 + Pmab	Positive	KRAS WT: 34.2	0.62	KRAS WT: 10.9	0.87	
							24.3	*p* = 0.009	10.1	*p* = 0.353	
					mFOLFOX6 + Cet		RAS WT: 41.3	0.63	RAS WT: 13.9	0.65	
							28.9	*p* = 0 .058	9.5	*p* = 0.029	
Bennouna et al. (2013)	NCT01126112	33	KRAS WT	First-line	Pmab	NA	7.1		4.3		9.1
VOLFI	NCT01328171	96	RAS WT	First-line	mFOLFOXIRI + Pmab	Positive	35.7	0.67	9.7	1.07	87.3
					mFOLFOXIRI		29.8	*p* = 0.12	9.7	*p* = 0.76	60.6
											*p* = 0.004
GONO	NCT01358812	37	RAS/BRAF WT	First-line	FOLFOXIRI + Pmab	NA			11.3		89.0
Pietrantonio et al. (2019)	NCT02476045	229	RAS WT	Maintenance after First-line	LV/5-FU + Pmab	Positive	18 months OS rate	1.13	10 months PFS rate	1.51	
					Pmab		66.4%	*p* = 0.60	59.9%	*p* = 0.009	
							62.4%		49.0%		
SAPPHIRE	NCT02337946	164	RAS WT	Maintenance after First-line	mFOLFOX6 + Pmab	Positive	8.1	0.9	9.1	0.93	
					5-FU/LV + Pmab		8.1		9.3		
PANAMA	NCT01991873	248	RAS WT	Maintenance after First-line	5-FU + Pmab	Positive	28.7	0.84	8.8	0.72	10.8
					5-FU		25.7	*p* = 0 .32	5.7	*p* = 0 .014	26
											*p* = 0.02
PICCOLO	ISRCTN93248876	460	KRAS WT	Second-line	IRI	Positive	10.9	1.01		0.78	
					IRI + Pmab		10.4	*p* = 0.91		*p* = 0.015	
20050181	NCT00339183	1186	KRAS WT	Second-line	Pmab + FOLFIRI	Positive	14.5	0.92	6.7	0.82	
					FOLFIRI		12.5	*p* = 0.37	4.9	*p* = 0.023	
WJOG 6510G	UMIN000006643	121	KRAS WT	Second-line	Pmab + IRI	Positive	14.85	0.66	5.42	0.64	
					Cet + IRI		11.53	*p* = 0.050	4.27	*p* < 0.001	
Van Cutsem et al. (2014)		463	NA	Third-line	BSC + Pmab	Positive		1	8 W	0.54	10.0
					BSC				7.3 W	*p* < 0.0001	0
Van Cutsem et al. (2016)		176	NA	Fourth-line	BSC + Pmab	NA	6.3 W		9.4 W		
20100007	NCT01412957	377	KRAS WT	Third-line	Pmab + BSC	Positive	RAS WT: 10.0	0.72	RAS WT: 5.2	0.45	31.0
					BSC		6.9	*p* = 0.015	1.7	*p* < 0.0001	2.3
Hecht et al. (2007)		148	EGFR (+)	Third-line	Pmab	NA	9 W		14 W		
GERCOR	NCT00655499	65	KRAS WT	Third-line	Pmab + IRI	NA	9.7 W		5.5 W		29.2

ITT, intention to treat; mOS, median overall survival; mPFS, median progression-free survival; ORR, objective response rate; EGFR, epidermal growth factor receptor; MT, mutation; WT, wild-type; NA, not applicable; Cet, cetuximab; Bev, bevacizumab; Pmab, Panitumumab; IRI, irinotecan; 5-FU, fluorouracil; BSC, best supportive care; LV, folinic acid; W, weeks.

#### 3.3.4 Maintenance treatment

The MACRO2TTD study (Aranda et al., 2018) confirmed insignificant differences in mPFS and mOS between continuation of the original regimen and maintenance treatment with cetuximab alone after induction therapy with mFOLFOX + cetuximab. Moreover, the MACBETH study (Cremolini et al., 2018a) suggested that cetuximab could be used for maintenance therapy in patients with RAS/RAF WT mCRC. However, current guidelines do not recommend the use of cetuximab for maintenance therapy.

#### 3.3.5 Second-line treatment

The BOND study (Cunningham et al., 2004) confirmed that cetuximab in combination with chemotherapy achieved a better therapeutic benefit than cetuximab alone in mCRC treatment, with increase in mOS (8.6 vs. 6.9 months, *p* = 0.48) and mPFS (4.1 vs. 1.5 months, *p* < 0.001). Cetuximab was approved by the FDA in 2004 as a second-line treatment for patients with mCRC. Subsequent studies (Sobrero et al., 2008; Ciardiello et al., 2016) have confirmed the therapeutic benefit of cetuximab in combination with chemotherapy over chemotherapy alone. Moreover, the UNICANCERPRODIGE18 study (Bennouna et al., 2019) showed that cetuximab had therapeutic benefits comparable to those of bevacizumab as second-line treatment.

#### 3.3.6 Third-line treatment

The NCT00079066 study (Jonker et al., 2007) demonstrated that cetuximab monotherapy resulted in higher OS and PFS in patients with refractory mCRC than the best supportive care (BSC) alone. Vincenzi et al. (2006) treated patients with mCRC using cetuximab + irinotecan as third-line chemotherapy, with an mPFS of 4.7 months and mOS of 9.8 months.

Rechallenge therapy means the reintroduction of targeted agent to which a tumor has already proven to be resistant (Tonini et al., 2013). Santini et al. (2012) performed cetuximab rechallenge as a third-line treatment for refractory mCRC and achieved promising results, with ORR of 53.8% [partial response (PR), 48.7%; complete response (CR), 5.1%] and mPFS of 6.6 months. Similarly, patients with KRAS WT mCRC rechallenged with cetuximab as third-line treatment exhibited positive results, with mPFS and OS of 2.4 and 8.2 months, respectively (Masuishi et al., 2020). A meta-analysis conducted by Mauri et al. (2019) showed that anti-EGFR rechallenge therapy yielded better therapeutic benefits than sequential and dose escalation therapies. Therefore, cetuximab could be used as a third-line treatment (Table 3).

### 3.4 Panitumumab

Panitumumab is an IgG2 monoclonal antibody that binds to EGFR, blocking the binding of VEGFR and VEGF and inhibiting cancer cell growth.

#### 3.4.1 First-line treatment

The PRIME study (Douillard et al., 2010) showed that treatment with FOLFOX4 + panitumumab significantly improved mPFS in patients with KRAS WT mCRC compared to that in patients with KRAS WT mCRC treated with FOLFOX4 alone (9.6 vs. 8.0 months, *p* = 0.02) but did not affect OS and was not significant in patients with KRAS MT. Moreover, the ASPECCT trial (Price et al., 2014) confirmed that panitumumab achieved results comparable to those of cetuximab in patients with KRAS WT mCRC. Based on these findings, panitumumab was approved by the FDA as a first-line treatment for patients with KRAS WT mCRC in 2014. A retrospective analysis of the PRIME study (Douillard et al., 2013) showed that all patients with RAS WT benefited from panitumumab treatment. Interestingly, the PEAK study (Schwartzberg et al., 2014) showed that panitumumab had better efficacy than bevacizumab in patients with KRAS/NRAS WT mCRC. Therefore, the FDA included NRAS WT mCRC as an indication for panitumumab treatment in 2017. Additionally, studies (Arnold et al., 2017; Boeckx et al., 2017; Peeters et al., 2018) have shown that panitumumab was more effective against RAS WT tumors located on the left side than those on the right side.

Furthermore, although treatment with mFOLFOXIRI + panitumumab did not significantly affect mOS and mPFS in patients with RAS WT mCRC, there was a significant increase (*p* = 0.004) in ORR (87.3% vs. 60.6%, *p* = 0.004) and metastasis resection-free recurrence survival (7.9 vs. 4.0 months) in the mFOLFOXIRI + panitumumab group compared to those in the mFOLFOXIRI group (Modest et al., 2019a). Similar results were found in the GONO study (Fornaro et al., 2013). Overall, panitumumab + FOLFOXIRI could be used for patients with RAS WT mCRC with metastases that require surgical resection.

#### 3.4.2 Maintenance treatment

The NCT02476045 study (Pietrantonio et al., 2019) showed that 5-Fu + panitumumab was more effective than 5-Fu only as maintenance treatment in patients with RAS WT mCRC, with 10-month PFS of 59.9% and 49.0% (*p* = 0.01) in the 5-Fu + panitumumab and 5-Fu groups, respectively. Additionally, there were no significant differences in PFS, OS, and RR between panitumumab + mFOLFOX6-and 5-Fu + panitumumab-treated patients with RAS WT mCRC (Munemoto et al., 2019). Moreover, the PANAMA study (Modest et al., 2022) confirmed that 5-Fu + panitumumab was a better maintenance treatment than 5-Fu alone. A retrospective analysis of the PRIME and PEAK studies confirmed that panitumumab was comparable to bevacizumab as a maintenance therapy (Modest et al., 2019b), indicating that panitumumab could be combined with other agents as a maintenance therapy for patients with RAS WT mCRC.

#### 3.4.3 Second-line treatment

The PICCOLO study (Seymour et al., 2013) showed that irinotecan + panitumumab was effective as a second-line treatment against KRAS WT mCRC than irinotecan only, as evidenced by a significant increase in PFS and RR in the panitumumab group; however, the OS was not significantly affected. Moreover, panitumumab had limited effects on patients with RAS MT. Similar results were obtained in the 20050181 study (Peeters et al., 2010; Peeters et al., 2014); therefore, panitumumab and cetuximab are sometimes used interchangeably as second-line treatments.

#### 3.4.4 Third-line treatment and beyond

Cutsem Eric Van Cutsem et al. (2007) reported that panitumumab monotherapy as a third-line treatment significantly increased (*p* < 0.0001) mPFS to 8 weeks in patients with mCRC compared to that in patients with mCRC treated with BSC only (7.3 weeks) but did not affect OS. Accordingly, panitumumab was approved by the FDA as a third-line treatment for patients with EGFR-positive mCRC in 2006. Similar results were observed in patients retreated with panitumumab + BSC (Van Cutsem et al., 2008). Overall, several studies have confirmed the efficacy of panitumumab monotherapy as a third-line treatment for mCRC (Hecht et al., 2007; Kim et al., 2016; Kim et al., 2018). Moreover, the GERCOR (André et al., 2013) and WJOG6510G (Sakai et al., 2020) studies both confirmed that panitumumab + irinotecan improved PFS in patients with KRAS WT mCRC but did not affect OS (Table 3).

### 3.5 Ramucirumab

Ramucirumab exerts its antitumor effects by specifically binding to VEGFR-2 and inhibiting tumor angiogenesis. The RAISE study (Tabernero et al., 2015) showed that treatment with FOLFIRI + ramucirumab increased mOS (*p* = 0.0219) and mPFS (*p* < 0.0005) to 13.3 and 5.7 months, respectively, compared with 11.7 and 4.5 months, respectively, in FOLFIRI-treated patients. Moreover, the treatment benefit of ramucirumab was superior to chemotherapy-only for both OS and PFS in any subgroup. Ramucirumab was approved as a second-line treatment for patients with mCRC by the FDA in 2015 (Table 3).

### 3.6 Fruquintinib

Fruquintinib is a VEGFR inhibitor that blocks neointimal growth associated with tumor proliferation and is a potent and highly selective small-molecule inhibitor of VEGFR1–3. The FRESCO study (Li et al., 2018) showed that fruquintinib + BSC treatment achieved considerable therapeutic effects in mCRC patients, irrespective of whether the patients were previously treated with anti-VEGF or anti-EGFR drugs, as evidenced by an increase (*p* < 0.001) in mOS (9.3 months) and mPFS (3.7 months) compared to those in mCRC patients treated with the BSC only (mOS, 6.6 months; mPFS, 1.8 months). Accordingly, fruquintinib was approved by the China National Medical Products Administration as a third-line treatment for patients with mCRC in 2018; however, fruquintinib has not been approved for use in other countries. Similarly, the results of FRESCO-2 study showed that fruquintinib treatment significantly improved (*p* < 0.001) mOS (7.4 months) and mPFS (3.7 months) compared to those of BSC treatment (mOS, 4.8 months; mFPS, 1.8 months) (Dasari et al., 2022). Key trials of HER2 targeted agents and TKIs in mCRC are summarized in Table 4.

**TABLE 4:** Major trials of HER2 mAbs and all TKIs in mCRC.

Trial name	Clinical number	Patiens (n)	Population	Treat line	Treatment	Result	mOS (months)	HR *P*-value	mPFS (months)	HR *p*-value2	ORR (%)
RAISE	NCT01183780	1072	NA	Second-line	Ramucirumab + FOLFIRI	Positive	13.3	0.844	5.7	0.793	
					Ramucirumab		11.7	*p* = 0.0219	4.5	*p* < 0.0005	
Jonker et al. (2007)	NCT01111604	153	NA	Second-line	mFOLFOX-6 + Ramucirumab	Negative	41.7 W	1.18	21.4 W	1.116	
					mFOLFOX-6		53.6 W		18.4 W	*p* = 0.623	
FRESCO	NCT02314819	416	NA	Third-line and beyond	Furoquinitinib + BSC	Positive	9.3	0.65	3.7	0.26	
					BSC		6.6	*p* < 0.001	1.8	*p* < 0.001	
FRESCO-2	NCT04322539	691	NA		Furoquinitinib + BSC	Positive	7.4	0.66	3.7	0.32	
					P + BSC		4.8	*p* < 0.001	1.8	*p* < 0.001	
CORRECT	NCT01103323	760	NA	Third-line and beyond	Regorafenib + BSC	Positive	6.4	0.77	1.9	0.49	
					BSC		5.0	*p* = 0.0052	1.7	*p* < 0.0001	
CONCUR	NCT01584830	204	NA	Third-line and beyond	Regorafenib placebo	Positive	8.8	0.55	3.2	0.31	
							6.3	*p* = 0.00016	1.7	*p* < 0.0001	
REVERCE	UMIN000011294		NA		Cet ± IRI after Regorafenib	Positive	17.4	0.61			
					Regorafenib after Cet ± IRI		11.6	*p* = 0.0293			
NCI-MATCH	NCT02465060	35	BRAF V600E MT		Darafenib + Trametinib	NA	28.6		11.4		
Corcoran et al. (2018)	NCT01750918	142	BRAF V600E MT		Pmab(P)+Darafenib(D)+Trametinib(T)	Positive	9.1		3.5		
					D + P		13.2		4.2		
					D + T + P		8.2		2.6		
					T + P						
Yaeger et al. (2015)	NCT01791309	12	BRAF V600E MT		Vemurafenib	NA	7.6		3.2		
Hong et al. (2020)	NCT01787500	19	BRAF V600E MT		Vemurafenib + IRI + Cet	NA			7.7		
BEACON	NCT02928224	605	BRAF V600E MT	Second-lineand beyond	Encorafenib + Binimetinib + Cet	Positive	9.3		4.5		
					Encorafenib + Cet		9.3		4.3		
					FOLFIRI ± Cet		5.9		1.5		
CodeBreaK100 (phase 1)	NCT03600883	42	KRAS	Thrid-line and beyond	sotorasib	Positive	12.8		4.0		20.0%
			G12C MT								
CodeBreaK100 (phase 2)	NCT03600883	62	KRAS G12C MT	Second-line and beyond	sotorasib	Positive	10.6		4.0		9.7%
HERACLES	EudraCT, number 2012-002128-33.	27	KRAS WT and HER2(+)	Second-line and beyond	Trastuzumab + Lapatinib	Positive					30.0
MyPathway	NCT02091141	57	HER2(+)	Second-line and beyond	Trastuzumab + Pertuzumab	Positive	11.5		2.9		32.0
HERACLES- B	NCT03225937	31	HER2(+)	Fourth-line and beyond	Trastuzumab + Pertuzumab	Positive			4.1		9.7
DESTINY-CRC01	NCT03384940	86	RAS WT and HER2 (+)	Third-line and beyond	Trastuzumab deruxtecan (DS-8201)	Positive	15.5(HER2 +++)		6.9 (HER2+++)		45.3
							7.3 (HER2 ++)		2.1 (HER2 ++)		
							7.7(HER2 +)		1.4 (HER2 +)		
MOUNTAINEER	NCT03043313	117	RAS WT and HER2 (+)	Fourth-line and beyond	Trastuzumab + Tucatinib	Positive	24.1		8.2		38.1%

mOS, median overall survival; mPFS, median progression-free survival; ORR, objective response rate; EGFR, epidermal growth factor receptor; HER2, human epidermal growth factor receptor 2; MT, mutation; WT, wild-type; NA, not applicable; Cet, cetuximab; Bev, bevacizumab; Pmab, Panitumumab; IRI, irinotecan; BSC, best supportive care; LV, folinic acid; W, weeks.

### 3.7 Regorafenib

Regorafenib is a TKI that acts on multiple targets, such as VEGFR and BRAF V600E. The CORRECT study (Grothey et al., 2013) showed that regorafenib + BSC improved mOS (6.4 months) and mPFS (1.9 months) in patients with mCRC compared to patients with mCRC treated with BSC only (mOS, 5 months; mPFS, 1.7 months). Accordingly, regorafenib was approved as a third-line treatment for patients with mCRC by the FDA in 2012. The CONCUR study (Li et al., 2015) demonstrated the benefits of regorafenib in Asian population. Regorafenib is the only monotherapy recommended by the NCCN guidelines for third-line therapy and further treatment (Table 4).

### 3.8 Encorafenib

Encorafenib primarily targets the BRAFV600E MT, which is commonly found in certain types of cancer. Additionally, it has inhibitory effects on JNK1–3, LIMK1–4, and STK36. The BEACON study (Kopetz et al., 2019) showed that encorafenib + binimetinib + cetuximab triple-agent treatment exerted considerable therapeutic effects in mCRC patients compared to those in the mCRC patients treated with FOLFIRI + cetuximab (control). However, subsequent studies showed insignificant differences in OS and mPFS between encorafenib + binimetinib + cetuximab and encorafenib + cetuximab groups (Tabernero et al., 2021). Accordingly, the NCCN guidelines recommend the use of encorafenib in combination with anti-EGFR as a second-line treatment in patients with BRAFV600E MT mCRC (Table 4).

### 3.9 Dabrafenib and trametinib

Dabrafenib specifically targets BRAF mutations (V600E and V600K), while trametinib selectively inhibits MEK1 and MEK2, key components of the RAS/RAF/MEK/ERK signaling pathway, resulting in the tumor growth suppression. The NCT01072175 study (Corcoran et al., 2015) showed that combined second-line treatment with dabrafenib (BRAF inhibitor) and trametinib (MEK inhibitor) increased mPFS to 3.5 months in patients with BRAF V600E MT mCRC, with PR observed in 12% of the patients and CR in one patient. Similarly, the NCT01750918 study (Corcoran et al., 2018) showed that panitumumab + dabrafenib + trametinib treatment increased RR (21%), mPFS (4.2 months), and mOS (13.2 months) in patients with BRAFV600E MT mCRC compared to those in patients with BRAFV600E MT mCRC treated with panitumumab + dabrafenib or panitumumab + trametinib. Therefore, the 2020 NCCN guidelines recommend that dabrafenib + trametinib could be combined with cetuximab or panitumumab as a second-line treatment for BRAFV600E MT mCRC. However, the 2021 and 2022 NCCN guidelines do not include dabrafenib and trametinib in combination with anti-EGFR for mCRC treatment (Table 4).

### 3.10 Vemurafenib

Vemurafenib is a targeted therapy that specifically inhibits the mutated BRAF form, BRAFV600E. A combination of vemurafenib and panitumumab has been shown to have a 100% tumor shrinkage rate, mPFS of 3.2 months, and mOS of 7.6 months in patients with BRAFV600E MT mCRC (Yaeger et al., 2015). Similarly, RR of 35% and mPFS of 7.7 months was obtained in patients with BRAFV600E MT mCRC treated with a combination of irinotecan, vemurafenib, and cetuximab (Hong et al., 2016). Additionally, the SWOGS1406 study (Kopetz et al., 2021) showed that irinotecan + cetuximab + vemurafenib treatment significantly increased PFS in patients with BRAFV600E MT mCRC compared to patients with BRAFV600E MT mCRC administered treatment regimens without vemurafenib. Moreover, FOLFIRI + cetuximab + vemurafenib treatment achieved an ORR of 81%, mPFS of 9.7 months, and mOS of 15.4 months in patients with BRAFV600E MT mCRC (Wang et al., 2022). Overall, these results confirmed that vemurafenib plus anti-EGFR can achieve significant efficacy in patients with BRAF MT mCRC; however, vemurafenib is yet to be approved for use in mCRC patients owing to shortage of relevant trials and limited number of enrolled patients (Table 4).

### 3.11 Sotorasib and adagrasib

Sotorasib and adagrasib are potent inhibitors of KRASG12C, specifically designed to target this mutation and act as antineoplastic agents. These agents selectively bind to and inhibit the mutant KRASG12C protein, offering potential therapeutic options for patients with KRASG12C MT cancers. In the phase 1 CodeBreaK100 study (Fakih et al., 2022) involving KRASG12C MT solid tumors, sotorasib treatment resulted in a median mPFS of 4.0 months and an ORR of 7.1% in the mCRC group. In the phase 2 study specifically conducted with mCRC patients, the ORR was 12.9%, with a mOS of 10.6 months and mPFS of 4.0 months (Hong et al., 2020). MRTX849 study (Klempner et al., 2022) showed that adagrasib + cetuximab mPFS was 6.9 vs. 5.6 months in adagrasib only group, and ORR was 46% vs. 19. The aforementioned studies have provided evidence of sotorasib and adagrasib efficacy in the treatment of mCRC. Nevertheless, additional clinical validation is required to further substantiate their practical clinical application.

### 3.12 Trastuzumab, pertuzumab, lapatinib and tucatinib

Trastuzumab and pertuzumab are monoclonal antibodies that specifically bind to different epitopes of the HER2 receptor, inhibiting HER2 signaling and enhancing immune-mediated destruction of tumor cells. In contrast, lapatinib is a TKI that targets both HER2 and EGFR receptors, effectively blocking their activation and downstream signaling pathways. The HERACLES study (Sartore-Bianchi et al., 2016) showed that trastuzumab + lapatinib treatment achieved an ORR of 30% in patients with KRAS WT and HER2-positive mCRC who previously underwent anti-EGFR therapy. Moreover, trastuzumab + pertuzumab treatment (HER2 antibodies) achieved an ORR of 32%, mPFS of 2.9 months, and mOS of 1.5 months in patients with HER2 positive mCRC (Meric-Bernstam et al., 2019) and an mPFS of 5.3 months in RAS WT subgroup. The HERACLES-B study (Sartore-Bianchi et al., 2020) showed that trastuzumab + panitumumab treatment achieved an ORR of 9.7% and mPFS of 4.1 months in patients with RAS WT and HER2-positive mCRC who had previously been treated with a third-line regimen.

Trastuzumab deruxtecan (DS-8201) is a novel antibody-drug conjugate with a humanized anti-HER2 antibody, cleavable peptide linker, and potent topoisomerase I inhibitor payload that has been confirmed to be effective in multiple solid tumors, including CRC (Tsurutani et al., 2020). The DESTINY-CRC01 study (Siena et al., 2021; Yoshino et al., 2023) treated HER2-positive mCRC patients with DS-8201 after two or more prior regimens. The patients were divided into three cohorts based on HER2 expression levels: cohort A (HER2-positive, IHC 3+ or IHC 2+/ISH+), cohort B (HER2 IHC 2+/ISH-), and cohort C (HER2 IHC 1+). The mOS in cohorts A, B, and C was 15.5, 7.3, and 7.7 months, respectively, while the mPFS was 6.9, 2.1, and 1.4 months, respectively. Notably, the ORR was observed only in cohort A, with a rate of 24%.

Tucatinib blocks proliferation and the phosphorylation of HER2 and its downstream effector, which is a TKI. MOUNTAINEER Trial (Strickler et al., 2022) rolled mCRC patients with RAS WT and HER2-positive which was treatment before but without anti-HER2, mOS was 24.1 months and mPFS was 8.2 months in the tucatinib + trastuzumab. As a result of the remarkable breakthrough in this study, the FDA granted accelerated approval in 2023 for the use of tucatinib + trastuzumab as a second-line treatment in HER2+ and RAS WT mCRC patients.

In light of the research findings mentioned above, the NCCN guidelines recommend trastuzumab in combination with either pertuzumab, lapatinib, tucatinib, or DS-8201 as second-line and beyond treatment options for patients with mCRC who have RAS WT and HER2-positive (Table 4).

## 4 Conclusion

Recent years have witnessed remarkable advancements in tumor research, driven by breakthroughs in sequencing technologies. These advancements have enhanced our understanding of tumors, including their genetic and genomic alterations. The knowledge provided a basis for personalized treatments, identification of new therapeutic targets, and improved diagnostic methods for different types of cancer. Particularly, the rapid progress in single-cell sequencing technology has revolutionized the field by enabling investigations at individual tumor cell level. This approach has provided unprecedented insights into tumor heterogeneity, allowing us to gain a better understanding of the diverse cellular composition within tumors and identify potential therapeutic targets. Furthermore, it has enhanced our understanding of the tumor microenvironment, including the distinct subpopulations and functions of different cells, as well as their intricate interactions. These advancements have particularly highlighted the crucial role of the tumor microenvironment in studying mechanisms of drug resistance.

**FIGURE 3:**
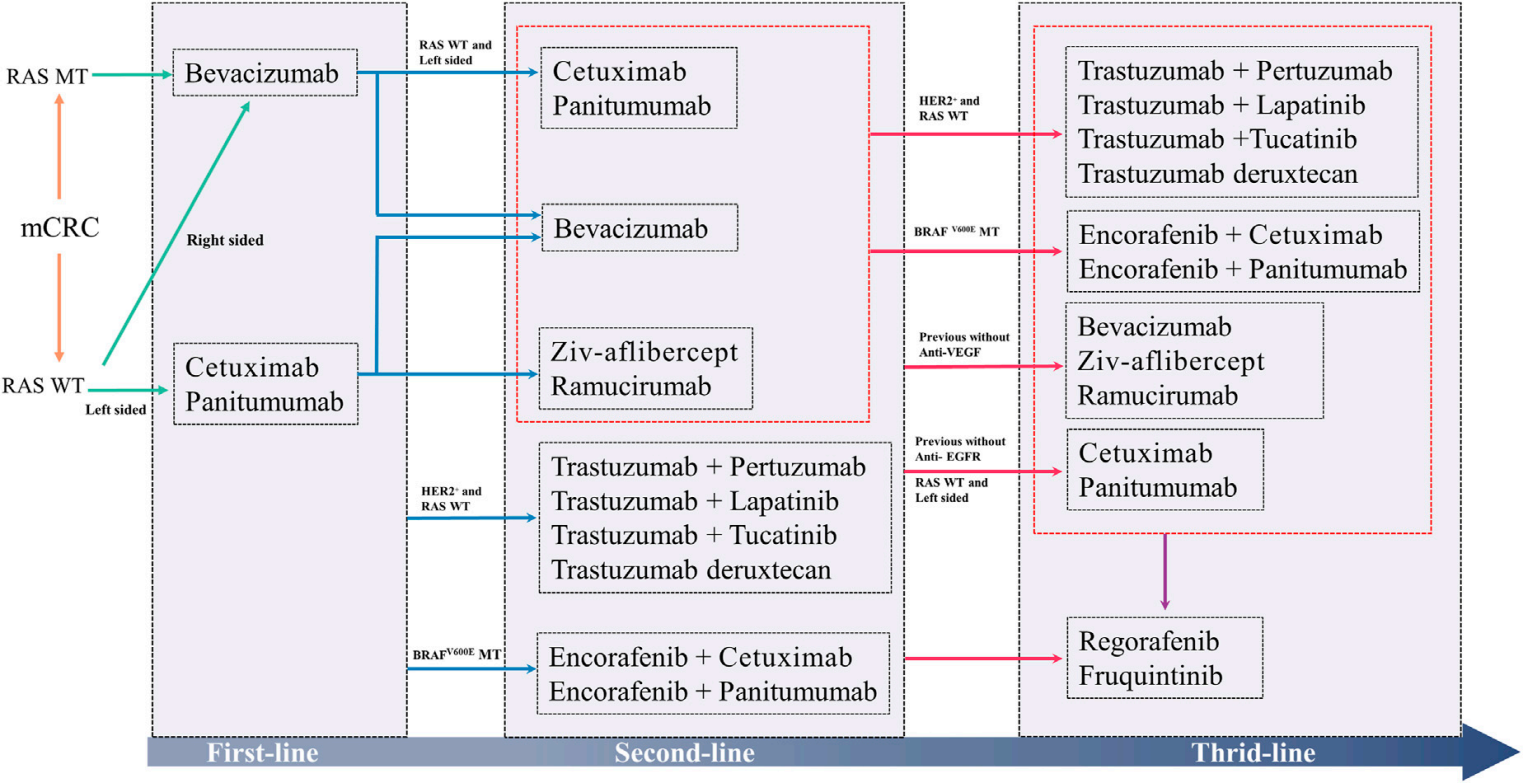
The current strategies for molecular targeted therapy in mCRC.

In the context of targeted therapy for tumors, significant improvements have been made in terms of CRC survival rates. The current strategies for targeted therapy in mCRC are summarized in Figure 3. However, several challenges remain. Drug resistance remains an inescapable obstacle, as the development of resistance in patients often goes unnoticed until disease progression occurs. Additionally, economic costs pose a major concern, as the need for testing multiple target markers further amplifies the financial burden on patients. Moreover, adverse drug reactions are a significant consideration, especially among vulnerable populations such as the elderly and children, given the varying tolerances to drug dosages. Striking a balance between achieving optimal efficacy and minimizing adverse effects remains an ongoing area of research.

Furthermore, the integration of immunotherapy and molecular targeted therapy has shown potential for further improvement of the survival rates of patients with tumors, including those with mCRC. However, combination of these therapies presents its own set of challenges requiring careful consideration. Co-administration of multiple drugs can potentially intensify adverse reactions, underscoring the importance of identifying the most effective combination regimen through thorough evaluation.

In summary, scientific progress has significantly enhanced our ability to combat CRC and other tumors. While obstacles and unanswered questions remain, the field of targeted therapy continues to advance, and it is anticipated that future clinical trials and research efforts will yield major breakthroughs, further increasing the survival rates and overall outcomes for cancer patients.

## Abbreviations

BSC, best supportive care; CRC, colorectal cancer; CRT, chemoradiotherapy; CR, complete response; Cap, capecitabine; DFS, disease-free survival; EGFR, epidermal growth factor receptor; FDA, United States Food and Drug Administration; HES, hairy and enhancer of split; HER, human epidermal growth factor receptor; mAbs, monoclonal antibodies; MAPK, mitogen-activated protein kinase; mCRC, metastasis colorectal cancer; mOS, median overall survival; mPFS, median progression-free survival; MT, mutation; mTOR, mammalian target of rapamycin; NCCN, National Comprehensive Cancer Network; NICD, Notch intracellular domain; ORR, objective response rate; OS, overall survival; PR, partial response; PI3K, phosphoinositide 3-kinases; RR, response rate; TKI, tyrosine kinase inhibitor; TRK, tropomyosin receptor kinase; TRK, tropomyosin receptor kinase; VEGF, vascular endothelial growth factor; WT, wild-Type; 5-FU, fluorouracil.
